# Comparative proteomics reveals the mechanism of cyclosporine production and mycelial growth in *Tolypocladium inflatum* affected by different carbon sources

**DOI:** 10.3389/fmicb.2023.1259101

**Published:** 2023-12-08

**Authors:** Junqi Wang, Meijie Liu, Chengzhi Mao, Sizhu Li, Jiabao Zhou, Yaqin Fan, Lizhong Guo, Hao Yu, Xiuqing Yang

**Affiliations:** Shandong Provincial Key Laboratory of Applied Mycology, School of Life Sciences, Qingdao Agricultural University, Qingdao, Shandong Province, China

**Keywords:** *Tolypocladium inflatum*, carbon sources, fructose, sucrose, cyclosporine A, mycelium

## Abstract

Cyclosporine A (CsA) is a secondary cyclopeptide metabolite produced by *Tolypocladium inflatum* that is widely used clinically as an immunosuppressant. CsA production and mycelial growth differed when *T. inflatum* was cultured in different carbon source media. During early fermentation, CsA was preferred to be produced in fructose medium, while the mycelium preferred to accumulate in sucrose medium. On the sixth day, the difference was most pronounced. In this study, high-throughput comparative proteomics methods were applied to analyze differences in protein expression of mycelial samples on day 6, revealing the proteins and mechanisms that positively regulate CsA production related to carbon metabolism. The differences included small molecule acid metabolism, lipid metabolism, organic catabolism, exocrine secretion, CsA substrate Bmt synthesis, and transcriptional regulation processes. The proteins involved in the regulation of mycelial growth related to carbon metabolism were also revealed and were associated with waste reoxidation processes or coenzyme metabolism, small molecule synthesis or metabolism, the stress response, genetic information or epigenetic changes, cell component assembly, cell wall integrity, membrane metabolism, vesicle transport, intramembrane localization, and the regulation of filamentous growth. This study provides a reliable reference for CsA production from high-efficiency fermentation. This study provides key information for obtaining more CsA high-yielding strains through metabolic engineering strategies.

## 1 Introduction

The application of cyclosporine A (CsA) is another great discovery in the exploration of secondary fungal metabolites since the discovery of penicillin (Survase et al., 2011; Kjærbølling et al., 2019). CsA inhibits the mixed lymphocyte response and contrasts with other immunosuppressants and cytostatic drugs with its weak myelotoxicity (Borel et al., 1976). Thus, CsA has been widely used in bone marrow immunotherapy and organ transplantation to inhibit chronic inflammatory reactions, HIV, and hepatitis C virus (Survase et al., 2011).

CsA is a non-ribosomal peptide (NRP) secondary metabolite mainly derived from the Ascomycota fungus *Tolypocladium inflatum* W. Gams 1971 (Rossman et al., 2017). *Tolypocladium inflatum* is widely produced in the pharmaceutical industry but more efficient production methods are needed to optimize the procedure. Current research has focused on obtaining better strains using chemical mutagenesis and optimizing medium using high throughput contrast tests. *Tolypocladium* strain VCRC F21 NRRL No. 18950 produces a high level of CsA in composition-optimized fermentation media and has been exploited for industrial production (Balaraman and Mathew, 2006). Diethyl sulfate-based mutagenesis was performed on the fungal strain *Tolypocladium inflatum* MTCC-3538. Liquid chromatography-mass spectrometry (LC–MS)-based high throughput medium has been used to optimize one of the mutants in 20 different media combinations to increase the CsA yield (Abrol et al., 2022).

The carbon source provides energy for cell growth and metabolism and is a key factor affecting fungal growth and the synthesis of secondary metabolites (Li et al., 2015). It has been reported that *Fusarium proliferatum* (Matsush.) Nirenberg cultured with sucrose as the only carbon source increases the production of fumonisin but fumonisin production is inhibited when using fructose as the carbon source (Jian et al., 2019). Replacing glucose with fructose in the initial fermentation medium is more conducive to the production of pneumocandin B_0_, the precursor of the antifungal drug caspofungin, and biomass accumulation (Zhang et al., 2020). Reduced growth, thinner hyphae, and visible injury were observed early during the cultivation of *Penicillium janczewskii* K.W. Zaleski in a fructose-containing medium, and the culture reached the maximum between days 12 and 15 (Pessoni et al., 2015). Some studies have attempted to replace CsA-producing strains with selected *Aspergillus terreus* Thom (FCBP58), and the CsA yield was effectively increased by optimizing the ratio of the carbon source to the nitrogen source in the medium (Tanseer and Anjum, 2011). However, few studies have analyzed how carbon sources affect the production of secondary metabolite CsA and its producing fungal mycelium growth.

Therefore, this aspect still needs further research. Some advancements in new technologies, like the various omics analyses, have become key tools to help us for these explorations and understand the molecular mechanisms that respond to changes in environmental conditions (Bi et al., 2018; Zhang et al., 2020). There are many successful studies here. The transcriptomic and proteomic analyses of the mycelium and fruiting bodies of *Cordyceps militaris* (L.) Fr. revealed differences in gene expression, including the cordycepin metabolism-related genes (Yin et al., 2012). The mechanism of the metabolic regulation and development of *Pleurotus ostreatus sensu* Stevenson fruiting bodies inhibited by a high CO_2_ concentration was revealed using proteomics analysis (Lin et al., 2022). The global protein expression profile of white *Hypsizygus marmoreus* (Peck) H.E. Bigelow mycelium under heat stress has been studied using a label-free comparative proteomics technique, and the molecular mechanism of mushroom heat stress response was explained (Xu et al., 2021). Comparative proteomic analysis techniques combined with protein-to-protein interaction networks have been used to study the differences in the intracellular transcription mechanisms of carbon-de-inhibited and wild-type *Pichia pastoris* (Guillierm.) Phaff strains fed with three carbon sources. The cluster modules of the differentially expressed proteins (DEPs) associated with the carbon sources were obtained, and the co-expression relationships between the DEPs have been constructed (Shi et al., 2020; Wang et al., 2022).

In this study, fructose and sucrose were used as carbon sources during fermentation of *T. inflatum*. We determined the CsA yield between the two carbon sources and analyzed the differences in protein expression and metabolic pathways by proteomics analysis. The proteins regulating CsA synthesis related to carbon metabolism were explored. This study provides additional candidate genes or proteins for modification through metabolic engineering strategies and affords new ideas for obtaining more CsA high-yielding strains.

## 2 Materials and methods

### 2.1 Sample material

Fructose and sucrose were used as carbon sources to prepare the liquid media called fructose medium and sucrose medium. Here was the recipe: D-(−)-fructose (Shanghai Sangon Biotech Co., Ltd., EINECS number: 200–333-3)/sucrose (Shanghai Sangon Biotech Co., Ltd., EINECS number: 200–334-9) 30 g/L, (NH_4_)_2_HPO_4_ 6 g/L, Yeast extract (Shanghai Sangon Biotech Co., Ltd., EINECS number: 232–387-9) 5 g/L, CaCl_2_·2H_2_O 1.32 g/L, MgSO_4_·7H_2_O 2.05 g/L, FeSO_4_·7H_2_O 27.4 mg/L, ZnSO_4_·7H2O 17.8 mg/L, CoCl_2_·6H_2_O 27.5 mg/L, CuSO_4_·5H_2_O 3.1 mg/L, ddH_2_O added to 1 L final volume. Adjusted pH to 5. The media were sterilized with moist heat at 115°C for 15 min (Yang et al., 2018).

*Tolypocladium inflatum* NRRL 8044 was grown on potato dextrose agar (BD Difco, Sparks, MD, United States) for 2 weeks. The impurities and mycelia were removed by filtrating through sterile degreasing cotton with a thickness of about 0.5 cm to obtain the spore suspension. The spore suspension was diluted to a final concentration of 10^8^ spores/mL. A 100 μL aliquot of the diluted spore suspension was removed and inoculated into 50 mL of liquid medium/250 mL conical flask at 25°C and 150 rpm in a constant temperature oscillating incubator for 3 days. The seed suspension was obtained after the incubation. Eight mL of the seed suspension was removed and inoculated into 250 mL of liquid medium/500 mL conical flask at 25°C at 150 rpm shaking for 9 days. Three biological replicates were used for each group.

The mycelia could be weighted and collected beginning on day 4. At the same time every day, 15 mL of culture broth including the fermentation product was removed and the mycelia were collected and placed in liquid nitrogen or stored at −80°C to determine hydrogen peroxide content, CsA production, total protein, and RNA.

### 2.2 Determination of CsA content

The Agilent 1,100 high-performance liquid chromatograph (Agilent Technologies Inc., Shanghai, China) was used to detect CsA, with a diode array UV detector. The separation was performed using the yuexu xtimate C18 column (particle size: 5 μm; column length: 4.6 × 250 mm); the sample size was 10 μL, and the detection wavelength was 210 nm. The parameter settings were column temperature of 40°C, mobile phase: A: water, B: acetonitrile (CAN), at a flow rate of 1 mL/min. The elution procedure was 0–28 min, 65–71.7% B; 28–34 min, and 71.7–65% B. The peak corresponding to CsA appeared at about 21 min (Yang et al., 2018).

The standard curve to determine the absolute weight of CsA was y = 2972.1 x - 286.44, R2 = 0.9985, where x is the absolute weight of CsA, unit: μg; y was the integrated peak area, and the units are mAU·s. The CsA standard was 98% pure (Aladdin).

### 2.3 Determination of accumulated biomass

A 5 mL aliquot of the fermentation products was thoroughly dehydrated at low temperature in a freeze-drying machine (Scientz-18 N/A, Ningbo Scientz Biotechnology Co., Ltd., Ningbo, China). The products were weighed to dryness to determine dry weight (DW).

### 2.4 Total protein acquisition and mass spectrometry detection

The proteome was detected using label-free LC–MS/MS Quantitative Proteomics Analysis technology (Benagen Co., Ltd., Wuhan, China). All protein extraction and digestion methods and the MS detection for total proteins were described in a previous study (Yang et al., 2021).

### 2.5 Protein identification and DEP screening

The total protein sequences from the raw files were screened out with a false discovery rate (FDR) < 0.05 for mapping to the *T. inflatum* genomic database (Bushley et al., 2013). Raw data were normalized by Proteome Discovery software suite version 2.0 (Thermo Fisher Scientific, San Jose, CA, United States). Firstly, missing values were supplemented by the k proximity method. Then, median standardization was executed on intensity data. After that, EdgeR was used to screen DEPs with |log_2_ (fold change)| > 1 and value of *p*<0.05. The protein expression difference of different samples was shown by a volcano plot constructed by TB tools.

### 2.6 Functional annotation of the DEPs

The DEPs were annotated and functionally enriched using Uniprot and Pfam. We also performed GO and KEGG analyses to annotate the DEPs. TBtools was used for the enrichment analyses.

The GO tool (http://geneontology.org; accessed on May 2023) was used and different terms were enriched. All terms were divided into three classes cellular components (CC), molecular functions (MF), and biological processes (BP).

The DEPs mapping to the KEGG pathways were retrieved by blasting against the KEGG database (https://www.genome.jp/kegg/pathway.html; accessed on May 2023). The pathways were attributed to several classes, such as Metabolism (A09100), Brite hierarchies (A09180), Genetic information processing (A09120), and Cellular processes (A09140).

### 2.7 Co-expression analysis of the DEPs

The String (search tool for the retrieval of interacting genes/proteins) database was used to accurately identify and annotate all co-expressed DEPs.^1^ The network of related DEPs in each sample was constructed using Cytoscape. The minimum required interaction score of confidence was 0.4. Proteins with high connectivity in the network were identified as hub DEPs and were believed to play core roles in co-regulating life processes. The DEPs that closely contacted the hubs were hub-surrounded DEPs, which assisted in determining the functionality of the hubs.

### 2.8 The proteomic data were validated by quantitative real-time PCR analysis

Total RNA and cDNA were prepared according to the description of the previous job (Yu et al., 2018). The threshold cycle values were normalized by the expression level of the 18S rRNA gene (Liu et al., 2018). RT-qPCR reactions were performed in a volume of 15 μL on a LightCycler® 96 Real-Time PCR System (F. Hoffmann-La Roche Ltd., Basel, Switzerland) with ChamQ Universal SYBR qPCR Master Mix (Sparkjade, Shandong, China) according to the manufacturer’s instruction. Expression levels were calculated according to the 2^–Δ Δ CT^ method.

## 3 Results and discussion

### 3.1 Changes in CsA yield and mycelium in fructose medium and sucrose medium, respectively

**Figure 1:**
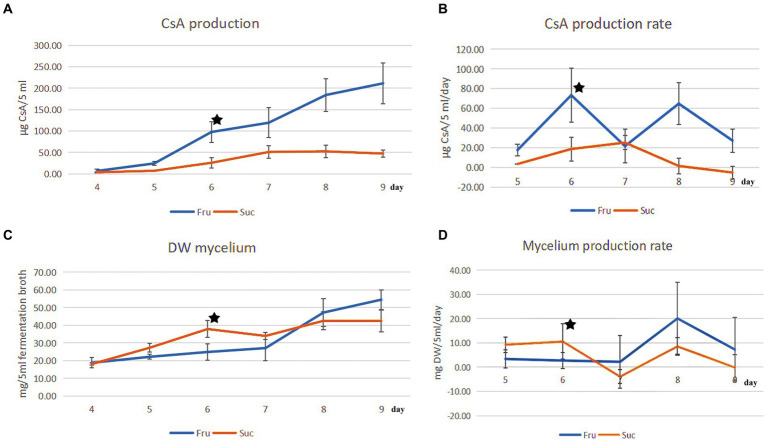
Comparison of CsA production and mycelial production from different carbon sources. **(A)** The change in the CsA yield from days 4 to 9 was cultured separately in fructose and sucrose media. **(B)** The CsA production rate from days 5 to 9. **(C)** The change in mycelial production corresponded to the change in CsA yield. **(D)** The mycelial production rate corresponded to the CsA production rate. ★: There were different priorities in CsA production and mycelium production on the 6th day.

CsA and mycelium production increased over time, and their production rates were different every day. The maximum CsA production rate in fructose medium was 73.38 μg CyA/5 mL fermentation broth/day (μg/5 mL/day) on day 6, and the rate of increase was the fastest. Compared with the rate on day 5 (17.60 μg/5 mL/day), the rate of increase was 316.93% [=(73.38–17.60)/17.60] (Figures 1A,B). However, mycelium production increased slowly in the fructose medium on day 6 at a production rate of 2.72 mg DW mycelium/5 mL fermentation broth (mg DW/5 mL) (Figures 1C,D).

However, the CsA yield and production rate in sucrose medium were much lower than those in fructose medium. On day 6, the CsA production rate was 18.71 μg/5 mL/day, which was 1/3.92 of that in fructose medium (Figures 1A,B). However, the mycelium production rate reached a maximum of 10.58 mg DW/5 mL on day 6, which was 3.89 times higher than the mycelium production rate in fructose medium (Figures 1C,D). CsA production, CsA production rate, mycelium production, and mycelium production rate were recorded (Supplementary Table S1).

**Figure 2:**
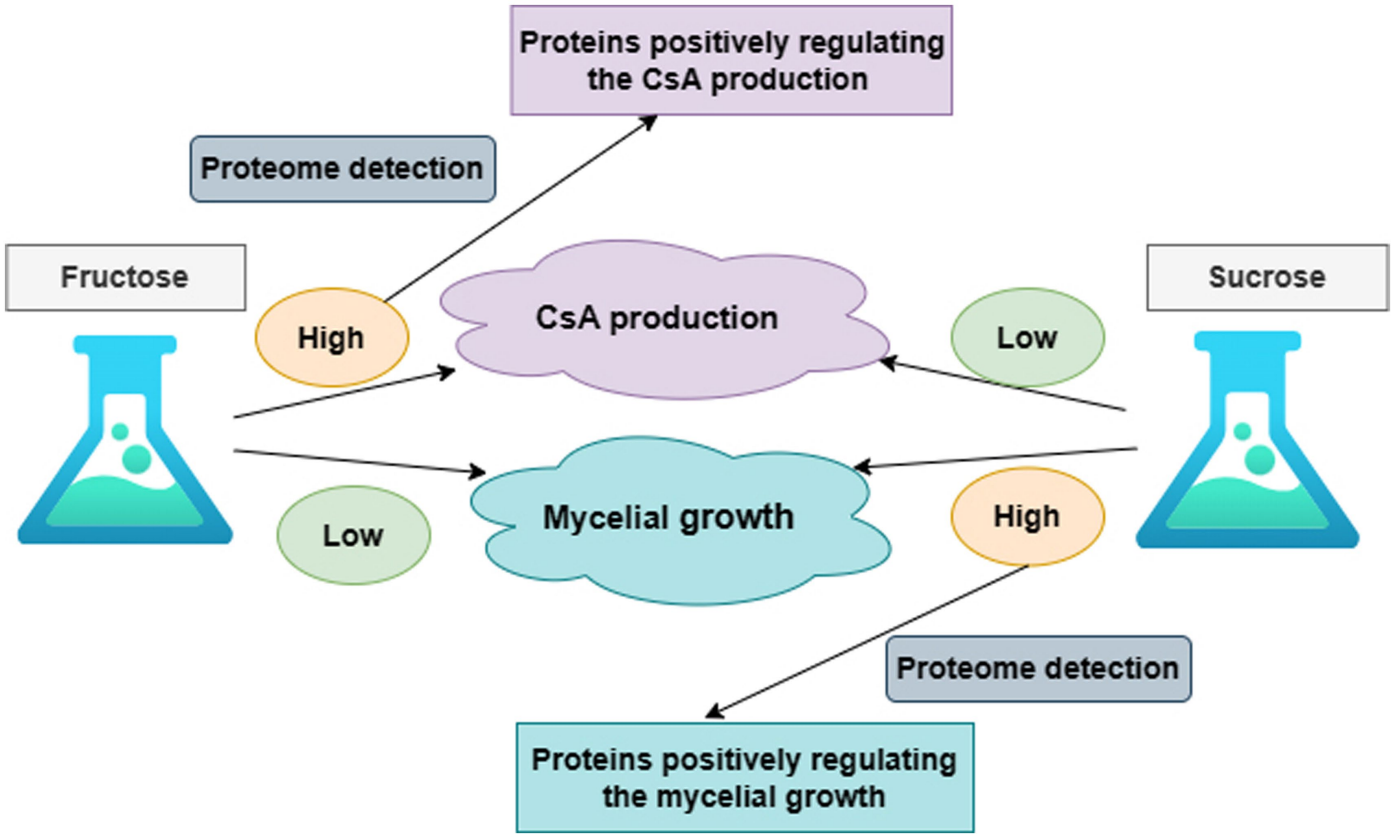
The overall research idea to illustrate the correlation between CsA production, mycelium growth, and protein expression.

It was known that the production of CsA and the accumulation of mycelium had different priorities in these two different media according to the above results. On day 6, there was a higher priority for the production of CsA in the fructose medium, at this point fructose became a better carbon source for promoting CsA production, and the relatively high expression protein in the fructose fermentation product was more likely to be positive regulatory for the production of cyclosporine. However, mycelium preferentially accumulated in the sucrose medium on day 6, so sucrose was a better carbon source suitable for mycelium growth and the highly expressed protein in the sucrose fermentation was more likely to be a positive regulatory protein promoting mycelial growth (Figure 2).

### 3.2 Label-free LC–MS/MS technology was used to enable the high-throughput proteomic analysis of the samples separately cultured in fructose medium and sucrose medium

**Figure 3:**
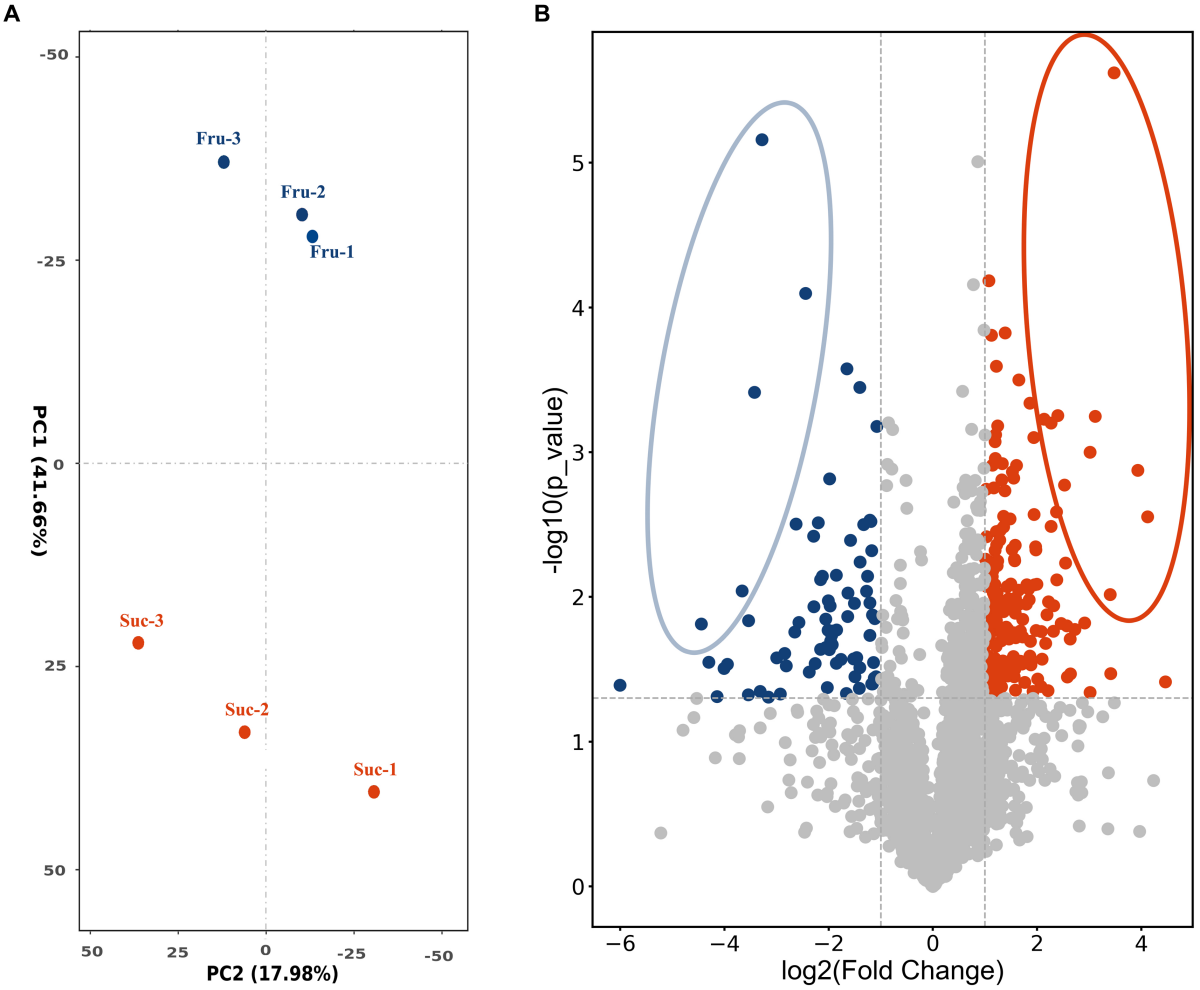
PCA analysis and volcano plot. **(A)** PCA plot shows the clustering of the samples based on their similarities. **(B)** The volcano plot shows the differential expression of the proteins in Fru and Suc. Each dot represents one protein. The horizontal coordinate measures the difference, and the vertical coordinates measure the significance of the difference. The highly expressed DEPs are blue in the Fru and red in the Suc. DEPs with relatively large and significant differences are circled.

To screen the positively regulating proteins related to carbon metabolism of CsA synthesis and mycelial growth, fresh mycelium obtained on day 6 was selected as the test sample according to the results and named “Fru” and “Suc.” The extracted total proteins were detected by label-free LC–MS/MS technology. A total of 3,227 proteins were identified and were median standardized (Supplementary Table S2). Principal component analysis indicated large differences between the groups and high similarities within the groups, indicating that the data were valid and could be used for further analysis (Figure 3A).

### 3.3 Screening of the DEPs

The DEPs were screened to compare the expression of Fru with Suc. A total of 244 DEPs were obtained and annotated (Supplementary Table S3-Total), including 74 highly expressed DEPs in the Fru (Supplementary Table S3-Fru) and 170 highly expressed DEPs in the Suc (Supplementary Table S3-Suc). The volcano plot shows the different effects of protein expression between the two groups and the distribution of the DEPs in each group. The different DEPs are circled and marked in yellow in the DEP list (Supplementary Table S3-Fru, Suc), and they may play a role in the trait formation of each group (Figure 3B). Four of the highly expressed DEPs, which are marked in green in the DEPs list (Supplementary Table S3-Fru), have been verified to be enzymes from the CsA biosynthetic gene cluster (Yang et al., 2018), including non-ribosomal peptide synthetase (NRPS, SimA, TINF00159), polyketide synthase (PKS, SimG, TINF00267), aminotransferase (SimJ, TINF00351), and ABC transporter (SimD, TINF00536). None of the CsA biosynthetic gene cluster proteins were highly expressed in Suc. These results further demonstrate that our analysis was accurate.

### 3.4 Functional analysis for the highly expressed DEPs in the Fru

**Figure 4:**
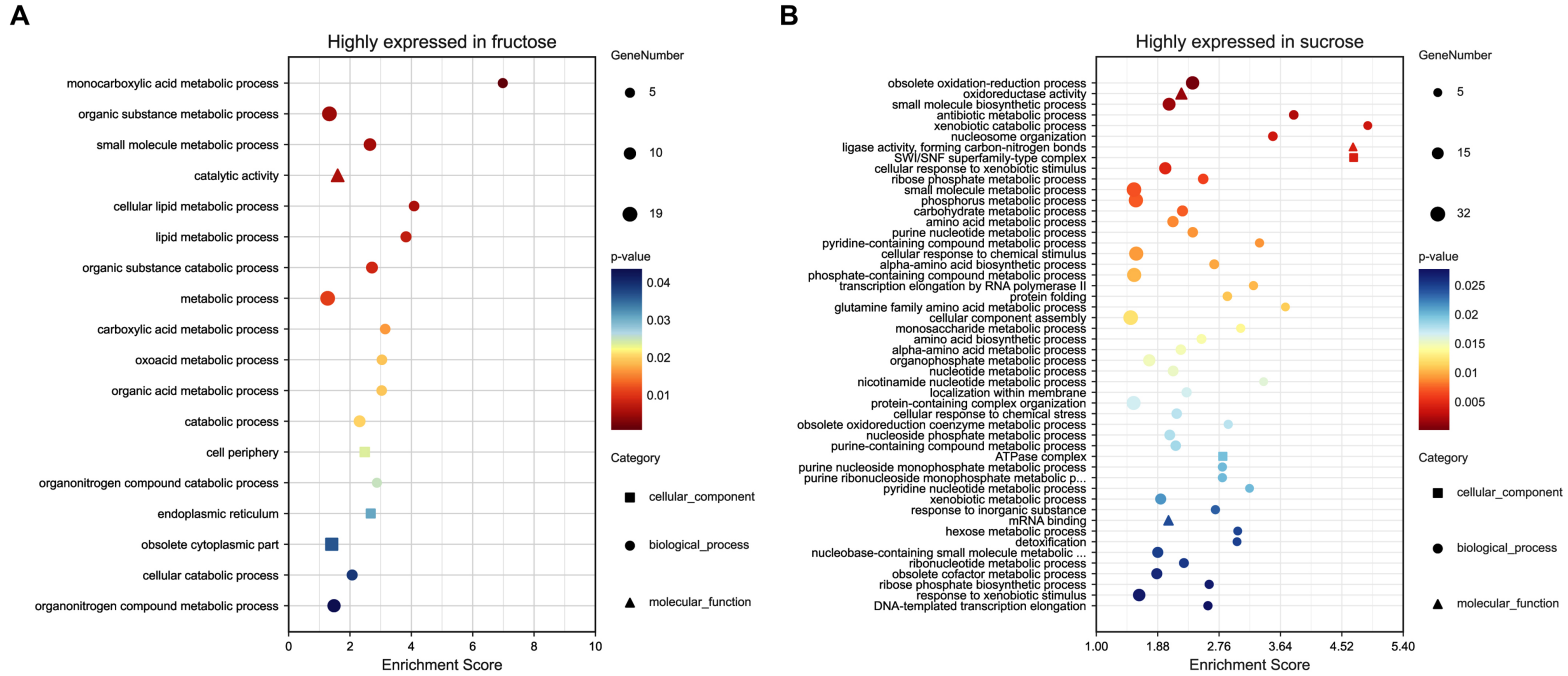
**(A)** GO analysis of the highly expressed DEPs in the Fru. **(B)** GO analysis of the highly expressed DEPs in the Suc. The GO terms were distributed into three classes, biological process (•), molecular function (▴), and cellular component (◼). The larger the graph, the more DEPs were involved. *p*-values ranged from 0 to 0.05, indicating warmer colors and more significant differences. The enrichment score (ES) is the maximum statistical value accumulated by the degree of association between each protein in the protein set and the phenotype, representing the degree of association between the whole protein set and the phenotype.

Eighteen terms (*p* < 0.05) were significantly enriched in the GO analysis, including the BP terms of small molecules, such as carboxylic acid, oxoacid, organic acid, metabolic processes, lipid metabolic processes (*p* < 0.01), cellular/organic substance catabolic processes, organonitrogen compound catabolic processes, (organic substance) and metabolic processes, including the CC terms of cell periphery, endoplasmic reticulum, and obsolete cytoplasmic part and the MF term of catalytic activity (*p* < 0.01). The DEPs annotations, expression ratios, and *p*-values are listed (Figure 4A; Supplementary Table S4-1).

**Figure 5:**
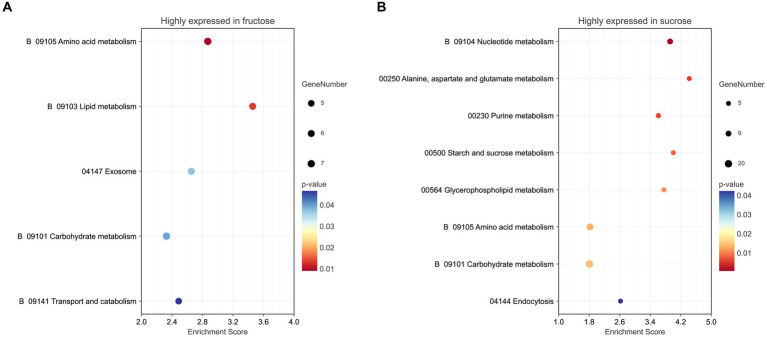
**(A)** KEGG pathway analysis of the highly expressed DEPs in the Fru. **(B)** KEGG pathway analysis of the highly expressed DEPs in the Suc. The color of the graph represents the value of *p* ranging from 0 to 0.05. The size of the graph represents the number of the included DEPs.

Five pathways (*p* < 0.05) were enriched in the KEGG pathway enrichment analysis. Three pathways were involved in metabolism, including amino acid metabolism (*p* < 0.01), lipid metabolism, and carbohydrate metabolism. The exosome pathway belonged to the Brite hierarchy. The last pathway was transport and catabolism, which belonged to the CP. The detailed KEGG information is listed (Figure 5A; Supplementary Table S5-1).

**Figure 6:**
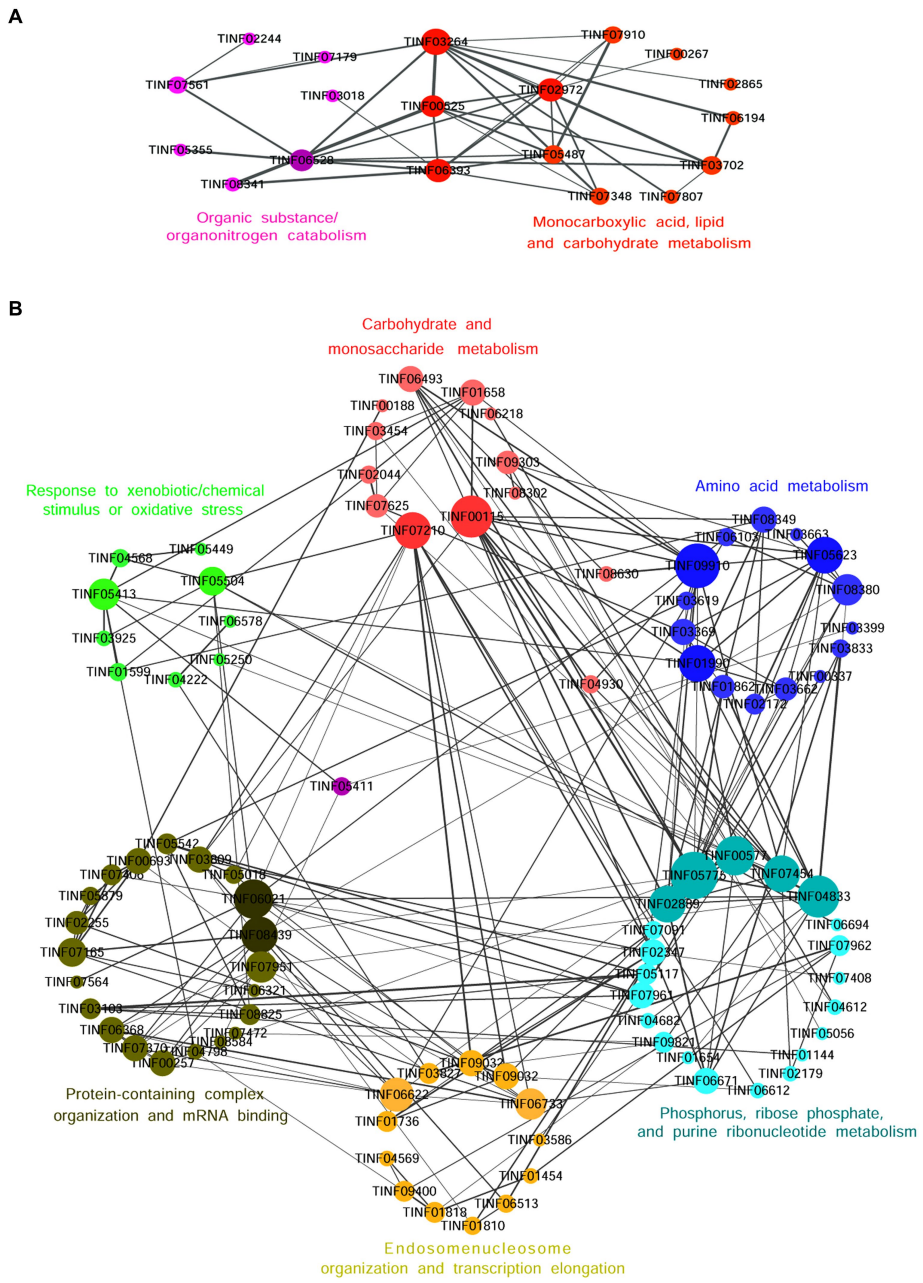
The co-expression network diagrams of the functionally related DEPs in the Fru **(A)** and the Suc **(B)**. Circles represent one DEP and size indicates the connectivity value of the proteins. The hub DEPs with high connectivity values are marked in deeper colors. The straight lines represent edges and the width of a straight line indicates the weighted value. The larger the weighted value between two DEPs, the closer their relationship.

Nineteen highly expressed DEPs were functionally related to Fru and connected in the network with the help of a co-expression analysis (Figure 6A). Twelve of these DEPs were related to small molecules, such as monocarboxylic acid, lipid, amino acid, and carbohydrate, metabolic processes (GO: 0044281, 0032787, 0006629, 0044255, 0019752, 0043436, 0006082, 0071704; KEGG: B 09103, B 09105, B 09101). The hub DEPs were aldehyde dehydrogenase (TINF03264), long-chain-fatty-acid-CoA ligase 1 (TINF02972), 2,4-dienoyl-CoA reductase [(3E)-enoyl-CoA-producing] (TINF06393), and malate synthase (TINF00525). Among them, polyketide synthase (TINF00267) was a major enzyme in the CsA gene cluster. The TINF02972 hub positively regulates CsA synthesis because it has a direct co-expression relationship with TINF00267. Ten of the DEPs were related to organic substances, particularly organonitrogen compounds, catabolic processes (GO: 1901575, 0009056, and 1,901,565), and transport and catabolism (KEGG: B 09141). The hub DEPs were TINF06393, TINF00525, TINF03264, and catalase (TINF06528). Notably, almost all DEPs involved in this network belonged to the obsolete cytoplasmic part (GO:0044444) or exosome (KEGG: 04147), indicating that these proteins were less relevant to primary metabolism, and more relevant to secondary metabolism, further reminding us that these proteins are related to the synthesis, metabolism, and transportation of secondary CsA metabolites. The DEPs involved in the network are listed and the hub DEPs are marked in red (Table 1).

**Table 1:** The highly expressed DEPs of Fru in a network.

Classification	Protein id	Functional annotation	Connective degree
Monocarboxylic acid,lipid and carbohydrate metabolism	TINF03264	Aldehyde dehydrogenase	10
	TINF02972	Long-chain-fatty-acid-CoA ligase 1	8
	TINF06393	2,4-dienoyl-CoA reductase [(3E)-enoyl-CoA-producing]	8
	TINF00525	Malate synthase	7
	TINF03702	glycerol kinase	5
	TINF05487	Acyl-CoA dehydrogenase	5
	TINF07348	methylmalonate-semialdehyde dehydrogenase (CoA acylating)	4
	TINF07910	Oxidoreductase, 2-nitropropane dioxygenase family, putative	4
	TINF06194	Aldolase	2
	TINF07807	Oxidoreductase	2
	TINF02865	Amidase family protein	1
	TINF00267	Polyketide synthase, putative	1
Organic substance/ organonitrogen catabolism	TINF03264	Aldehyde dehydrogenase	10
	TINF06393	2,4-dienoyl-CoA reductase [(3E)-enoyl-CoA-producing]	8
	TINF00525	Malate synthase	7
	TINF06528	Catalase	7
	TINF07561	Glutathione transferase (Gto1), putative	4
	TINF08341	Allantoicase	2
	TINF02244	Glutathione hydrolase	1
	TINF03018	Short chain dehydrogenase/reductase	1
	TINF05355	Phosphorylcholine phosphatase	1
	TINF07179	Alpha-mannosidase	1

The hub proteins were marked in yellow.

**Figure 7:**
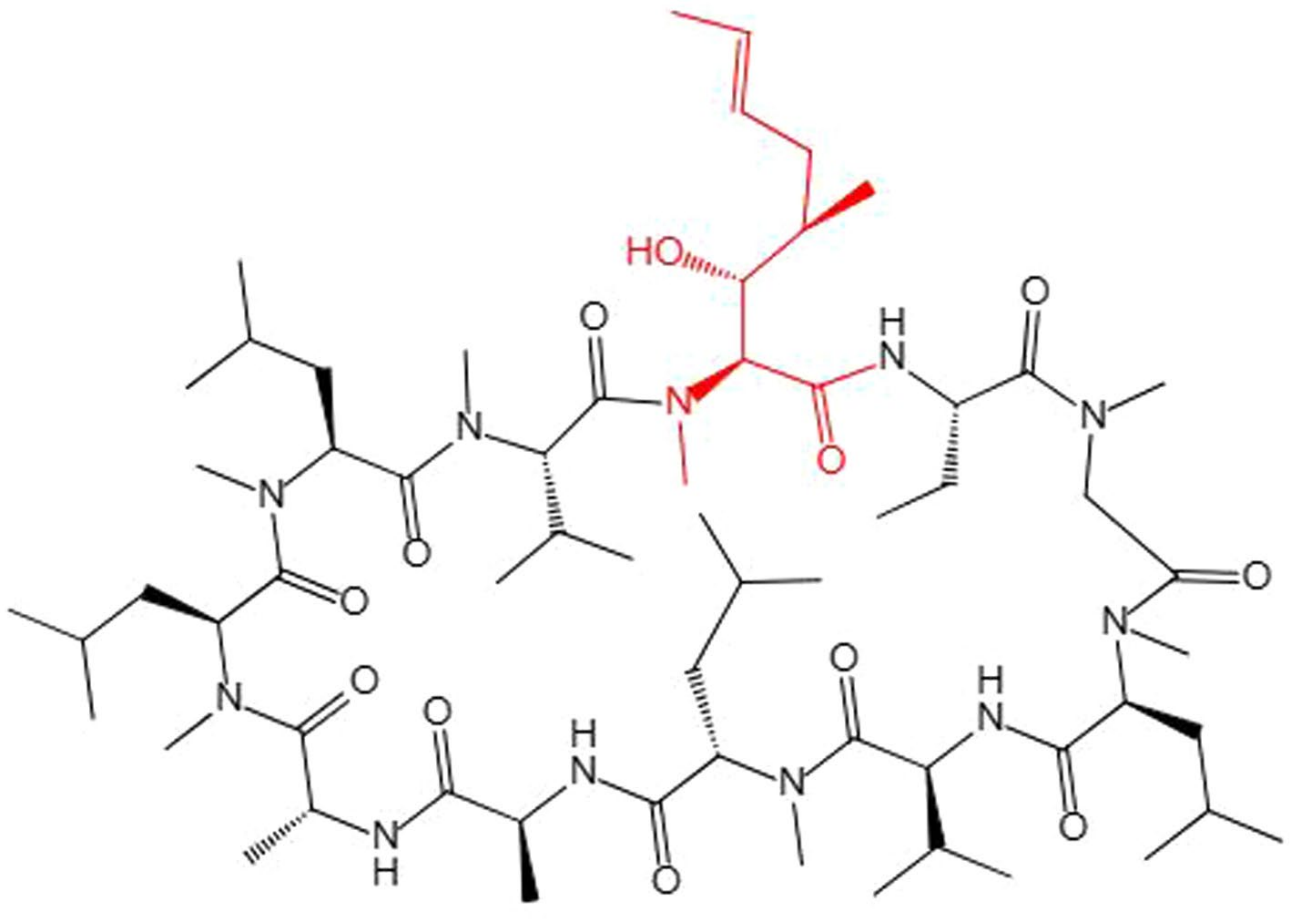
The chemical structure of CsA. MeBmt is red.

Many studies have improved the CsA yield. These mostly rely on the traditional method of mutagenesis and screening of high-yielding strains, but results using this method are random and counterproductive (Domratcheva et al., 2018). Although filamentous fungi could undergo some natural mutations as they grow, such as low-frequency gene recombination could be produced by parasexuality, some recombinants could be obtained for screening strains with a higher yield of secondary metabolites to a certain extent, but this evolutionary speed is not as fast as bacteria, which is far from meeting the needs of production (Meyer, 2008). CsA is a secondary cyclopeptide metabolite (Zhang et al., 2017) composed of 11 amino acids (Molnár et al., 2010). CsA synthesis requires the unusual amino acid (4*R*)-4-[(*E*) -2-butenyl]-4-methyl-*L*-threonine (Bmt) as a substrate (Kirihata et al., 1995), and MeBmt (partially methylated Bmt) was marked out (Figure 7). However, CsA chemical synthetic methods are mostly limited by the difficulty of obtaining Bmt, because Bmt does not exist in natural medium, and its yield is very low in Bmt-producing fungi. In addition, Bmt is difficult to isolate and purify from complex products. Some researchers have been working to obtain MeBmt through a chemical synthesis approach (Rolt et al., 2019). However, this method involves many steps, many intermediate products, and a low final yield, and is difficult to put into industrial production.

The full biosynthetic mechanism of CsA has been analyzed at the genetic level. The CsA biosynthetic gene cluster has been suggested to include 12 genes encoding enzymes, including NRPS (SimA, TINF00159) responsible for assembling the 11 amino acid substrates of CsA and PKS (SimG, TINF00267) to mediate Bmt production (Yang et al., 2018). Many attempts have been made to obtain high-yielding engineered strains by expressing important secondary metabolites genes or gene clusters in a heterologous host that are produced at low levels by the original strain (Lan et al., 2019). However, NRPS and PKS are usually abundant in filamentous fungi, so cloning and processing large DNA fragments or complete gene clusters to achieve their hetero-expression is a challenge (Nielsen et al., 2019). Mining more genes or proteins that positively regulate CsA synthesis from several perspectives will indicate the direction to obtain engineered strains with higher CsA yields and provide more space for genetic manipulation.

In addition to these proteins analyzed, other proteins may play vital roles in the regulation of CsA synthesis. It has been confirmed that cytochrome P450 (SimI, TINF00470) and aminotransferase (SimJ, TINF00351) are involved in Bmt synthesis (Yang et al., 2018). Two DEPs highly expressed in Fru were functionally similar. Copper radical oxidase (TINF06995) is a biocatalyst for the selective oxidation of primary alcohols to aldehydes and has a wide range of specificities for aliphatic compounds, which have a similar function to TINF00470 (Cleveland et al., 2021). The other DEP was an aminotransferase (putative, TINF08869), which was the same as TINF00351.

Overexpressing the basic leucine zipper (bZIP) transcription regulator (SimL, TINF00394), which directly regulates the expression of the CsA gene cluster, improves CsA production (Yang et al., 2018). Interestingly, three highly expressed DEPs regulated DNA transcription in Fru. The Myb transcription factor (TINF04891), which can be activated by bZip transcription factors, participated in transducing the aerial stimulus signal. Both factors jointly activate a C(2)H(2) zinc finger transcription factor, which plays a central role in fungal growth and virulence (Garzia et al., 2010; Lee et al., 2021). TINF04891 may participate in the regulation of CsA biosynthesis. Another protein containing the critical features of the tristetraprolin zinc finger domain has been linked to the control of pheromone signal transduction and the coordination of mitosis (Cuthbertson et al., 2008). The CCCH zinc finger DNA binding protein (TINF00841) may regulate the CsA biosynthetic process. The transcriptional activator ALCR, which is a transcriptional activator in the ethanol utilization pathway of filamentous fungi, is a DNA-binding protein with a helix-turn-helix structure (Kulmburg et al., 1992). The helix-turn-helix domain-containing protein (TINF02756) may also be a transcriptional activator of CsA biosynthesis.

The ABC transporter (SimD, TINF00536) in the CsA gene cluster may facilitate the production of more CsA and increase tolerance to the fungus by transferring CsA to reduce its concentration (Yang et al., 2018). The WSC domain-containing protein (TINF08949) may have a similar function as SimD because it localizes in the vacuoles and cell wall/membrane of the filamentous fungus *Beauveria bassiana* and, hence, has been linked to cell membrane- and vacuole-related cellular events (Tong et al., 2019). The short-chain dehydrogenase/reductase SDR (TINF06213, TINF03230) could also enhance fungal tolerance to secondary metabolites by the non-toxic transformation of secondary metabolites (Xing et al., 2021). It may also participate in CsA transformation.

### 3.5 Highly expressed DEPs in Suc

Industrial fermentation to produce secondary metabolites often requires a segmented culture of engineered fungi. More secondary metabolites are produced when the mycelium reaches a particular abundance. A previous study was aimed at optimizing the culture medium for biomass production and phenolic compounds using *Ganoderma lucidum*. The culture was optimized in two stages. After the maximum biomass production was reached under optimal conditions, more phenolic compounds were obtained (Zárate-Chaves et al., 2013). We analyzed the highly expressed DEPs in Suc medium, which are mostly involved in the regulation of *T. inflatum* mycelial growth.

Seventy-four terms (*p* < 0.05) were significantly enriched in the GO analysis, including the BP terms of obsolete oxidation–reduction processes and obsolete oxidoreduction coenzyme metabolic processes, small molecules, such as antibiotic and pyridine-containing compounds, biosynthetic or metabolic processes (*p* < 0.01), cellular response to xenobiotic/chemical stimulus/stress, response to inorganic substance/oxidative stress and detoxification, xenobiotic catabolic/metabolic processes, obsolete cofactor metabolic processes, ribose phosphate metabolic processes, phosphorus metabolic processes, nucleotide metabolic processes, nucleosome organization (*p* < 0.01), DNA conformational changes, transcription elongation, amino acid biosynthetic/metabolic processes, carbohydrate catabolic/biosynthetic processes, cellular component assembly/organization, localization within membranes, negative regulation of cellular component organization, and the regulation of filamentous growth. Additionally including the MF terms oxidoreductase activity (*p* < 0.01), ligase activity, forming carbon-nitrogen bonds (*p* < 0.01), mRNA binding, unfolded protein/protein binding, isomerase activity, including the CC terms SWI/SNF superfamily-type complex (*p* < 0.01), ATPase complex, cytosol, and endosome (Figure 4B; Supplementary Table S4-2).

Eight KEGG pathways (*p* < 0.05) were enriched. Seven pathways were involved in metabolism, including nucleotide metabolism (*p* < 0.01), amino acid metabolism, alanine, aspartate, and glutamate metabolism (*p* < 0.01), purine metabolism (*p* < 0.01), carbohydrates, such as starch and sucrose, metabolism, glycerophospholipid metabolism, and endocytosis in CP (Figure 5B; Supplementary Table S5-2).

Ninety-three DEPs were connected to a network (Figure 6B; Table 2). Thirteen were related to carbohydrate (GO: 0005975, 0016051, 0016052; KEGG: B 09101), monosaccharide (GO: 0005996), hexose (GO: 0019318), or starch and sucrose (KEGG: 00500) metabolic processes. The hub DEPs were phosphoglycerate kinase (TINF00115) and SNF2-family ATP-dependent chromatin remodeling factor snf21 (TINF07210). Six DEPs were related to carbon metabolism, TINF00115, ribose-phosphate diphosphokinase (TINF04682), serine/threonine-protein kinase cot-1 (TINF06493), dihydrolipoyl dehydrogenase (TINF09910), glycerol-3-phosphate dehydrogenase [NAD(+)] (TINF01599), and phosphoenolpyruvate carboxykinase (ATP) (TINF09303), (Supplementary Figure S1). Fifteen participated with amino acids, such as alanine, aspartate, and glutamate, metabolic processes (GO: 0006520, 0008652, 1,901,605, 1,901,607; KEGG: B 09105, 00250). The hub DEPs were TINF09910, glutamine synthetase (TINF01990), and glutamate dehydrogenase (TINF05623). Nine DEPs were related to amino acid biosynthesis, including TINF01990, TINF04682, TINF00115, TINF06493, asparagine synthetase (TINF01862), aconitate hydratase, mitochondrial (TINF03619), glutamate-5-semialdehyde dehydrogenase (TINF03662), imidazole glycerol phosphate synthase hisHF (TINF03833), and threonine synthase (TINF08349) (Supplementary Figure S2). Interestingly, TINF04682, TINF00115, and TINF06493 were closely related to carbon metabolism and amino acid biosynthesis. Twenty-one DEPs participated in phosphorus (GO: 0006793, 0006796; KEGG: 00564), ribose phosphate (GO: 0009123, 0009259, 0046390, 0019693, 0009161; KEGG: B 09104), and purine ribonucleotide (GO: 0009150, 0009126, 0009167, 0009199; KEGG: 00230) metabolic processes. The hub DEPs were adenylosuccinate lyase (TINF00577), GMP synthase [glutamine-hydrolyzing] (TINF04833), phospho ribosylformyl glycinamidine synthase (TINF02889), CTP synthase (TINF05775), and glycerol-3-phosphate dehydrogenase [NAD(+)] (TINF07454). Thirteen DEPs were components of the endosome (GO: 0005768) or enzymes contributing to transcription elongation (GO: 0006354, 0006368), nucleosome organization (GO: 0034728), and protein-DNA complex subunit organization (GO: 0071824). The hub DEPs were FK506-binding protein (TINF06622) and protein arginine N-methyltransferase 1 (TINF06733). Interestingly, another three DEPs from the network were associated with nuclear changes or transcription. The nuclear movement protein nudC (TINF04353) is required for nuclear migration during vegetative growth and development (Xiang et al., 1995). The polybromo-1 (putative, TINF04750) coordinates key features common to all remodeling complexes, including chromatin localization, recruitment of protein subunits, and changes in chromatin architecture (Thompson, 2009). The C2H2 transcription factor RfeC (TINF01322) has broad regulatory roles in various fungal growth and developmental processes, conidiation, and the abiotic stress response in eukaryotes (Chen et al., 2020). Twenty-two DEPs were related to protein-containing complex organization or assembly (GO: 0043933, 0065003) and mRNA binding (GO: 0003729). The hub DEPs were the eukaryotic translation initiation factor subunit eIF-4F, putative (TINF06021), nucleolar protein 58 (TINF08439), and WD repeat-containing protein 36 (TINF07951). TINF07951 and TINF08439 were related to ribosome biogenesis, and elongation factor 2 (TINF08620) was related to ribosome function (Supplementary Figure S3). Eleven DEPs were related to response to xenobiotic stimulus/inorganic substance/chemical stimulus (GO: 0009410, 0010035, 0042221, 0051716, 0070887, 0071466), xenobiotic catabolic processes (GO: 0042178), response to oxidative stress (GO: 0034599, 0055114), and detoxification (GO: 0098754). The hub DEPs were TINF07951, glutathione peroxidase (TINF05413), and mitochondrial protein import protein MAS5 (TINF05504). In addition, three highly expressed proteins from the network were associated with oxidative stress. Survival factor 1 (TINF07979) was involved in coping with reactive oxygen species, which promote survival under conditions of oxidative stress in *S. cerevisiae* (Yu et al., 2019). The non-specific serine/threonine protein kinase (TINF06713) is involved in energy flux and protein synthesis. Deleting this protein increases the sensitivity of yeast cells to oxidative stress (H2O2 treatment) and partially inhibits cell growth (Huang et al., 2014). Thioredoxin (TINF01860) is an enzyme comprising the thioredoxin system, which participates in resistance to oxidative stress (Missall and Lodge, 2005). Another two DEPs from the network participate in signal transduction in response to various abiotic stressors. Elongation factor 2 (TINF08620) could potentially be phosphorylated by the Rck2 kinase in response to environmental stress (Bartish et al., 2007). The AMP-activated protein kinase glycogen-binding domain-containing protein (TINF02240) is activated under conditions of nutrient or metabolic stress (Wiatrowski et al., 2004). Mitogen-activated protein kinases (MAPKs) are a group of serine–threonine protein kinases that are activated by different extracellular stimuli, such as cytokines, neurotransmitters, hormones, cellular stress, and cell adhesion. TINF01599, TINF06713, TINF07454, and the phosphotransmitter protein Ypd1 (putative, TINF01654) participated in the MAPK signaling pathway (Supplementary Figure S4).

**Table 2:** The highly expressed DEPs of Suc in a network.

Classification	Protein id	Functional annotation	Connective degree
Phosphorus,ribose phosphate,and purine ribonucleotide metabolism	TINF05775	CTP synthase	14
	TINF04833	GMP synthase [glutamine-hydrolyzing]	12
	TINF00577	Adenylosuccinate lyase	11
	TINF02889	phosphoribosylformylglycinamidine synthase	10
	TINF07454	Glycerol-3-phosphate dehydrogenase [NAD(+)]	10
	TINF06671	Phosphoribosylaminoimidazole carboxylase	6
	TINF07961	Cytochrome b-c1 complex subunit Rieske, mitochondrial	6
	TINF02347	Histone chaperone	6
	TINF05117	Cytochrome c oxidase subunit 6, mitochondrial	4
	TINF07962	GTPase-activating protein	4
	TINF09821	1-acyl-sn-glycerol-3-phosphate acyltransferase	4
	TINF07091	DUF1750 domain protein	3
	TINF04612	UDP-N-acetylglucosamine pyrophosphorylase	2
	TINF06612	RNA polymerase II transcription elongation factor Ctr9, putative	2
	TINF02179	choline-phosphate cytidylyltransferase	2
	TINF04682	ribose-phosphate diphosphokinase	2
	TINF06694	Hsp90 chaperone protein kinase-targeting subunit	1
	TINF07408	Protein phosphatase PP2A regulatory subunit	1
	TINF01144	V-type proton ATPase subunit F	1
	TINF05056	Kynurenine formamidase	1
	TINF01654	Phosphotransmitter protein Ypd1, putative	1
Endosome, nucleosome organization and transcription elongation	TINF06622	FK506-binding protein	9
	TINF06733	Protein arginine N-methyltransferase 1	8
	TINF04310	SWI-SNF complex subunit (BAF60b), putative	6
	TINF09032	Ran-specific GTPase-activating protein 1, putative	6
	TINF09400	Protein transport protein BOS1	4
	TINF01736	Transcription regulator BDF1, putative	4
	TINF01818	SNARE protein Snc2, putative	4
	TINF03827	Guanyl-nucleotide exchange factor (Sec7), putative	4
	TINF01810	Histone H1-binding protein	3
	TINF06513	DNA replication licensing factor MCM7	3
	TINF04569	t-SNARE	2
	TINF01454	Zinc finger protein gcs1	2
	TINF03586	Leukotriene A4 hydrolase	1
Protein-containing complex organization and mRNA binding	TINF06021	Eukaryotic translation initiation factor subunit eIF-4F, putative	11
	TINF08439	Nucleolar protein 58	10
	TINF07951	WD repeat containing protein 36	8
	TINF07165	Small nuclear ribonucleoprotein SmB, putative	7
	TINF06368	Nucleolin protein Nsr1, putative	6
	TINF00257	U3 small nucleolar RNA associated protein	6
	TINF00693	Pre-mRNA processing splicing factor, putative	6
	TINF07370	Protein PUF6, putative	6
	TINF03809	Replication protein A subunit	6
	TINF02255	mRNA splicing factor (Prp17)	5
	TINF03103	Cytochrome c oxidase polypeptide VIb	4
	TINF07406	Splicing factor U2AF subunit	4
	TINF05542	Mitochondrial export translocase Oxa1, putative	4
	TINF08825	Mitochondrial outer membrane translocase complex, subunit Tom22	4
	TINF05879	Pre-mRNA splicing factor CWC21	3
	TINF05018	Nuclear cohesin complex subunit (Psc3), putative	3	
	TINF04798	Endoplasmic reticulum transmembrane protein	2
	TINF06321	C2H2-type domain-containing protein	1
	TINF08584	Uncharacterized protein	1
	TINF07472	Proteasome component	1
	TINF07564	Topisomerase II associated protein	1
Response to xenobiotic/chemical stimulus or oxidative stress	TINF05413	Glutathione peroxidase	8
	TINF05504	Mitochondrial protein import protein MAS5	7
	TINF01599	Catalase	3
	TINF04568	Oxidoreductin	3
	TINF04222	Zinc homeostasis factor 1	2
	TINF03925	Monothiol glutaredoxin-5	2
	TINF05449	Protein disulfide-isomerase tigA	1
	TINF05250	Lysine decarboxylase-like protein	1
	TINF06578	Serine/threonine-protein kinase cot-1	1
Carbohydrate and monosaccharide metabolism	TINF00115	Phosphoglycerate kinase	12
	TINF07210	SNF2-family ATP dependent chromatin remodeling factor snf21	10
	TINF01658	1,4-alpha-glucan-branching enzyme	6
	TINF06493	Ribulose-phosphate 3-epimerase	6
	TINF07625	Alpha,alpha-trehalose phosphate synthase subunit TPS3	5
	TINF09303	Phosphoenolpyruvate carboxykinase (ATP)	5
	TINF03454	Glycogenin	3
	TINF02044	Trehalase	3
	TINF00188	TINF00188	1
	TINF08302	nitric oxide dioxygenase	1
	TINF06218	UDP-glucose 4-epimerase	1
Amino acid metabolism	TINF09910	Dihydrolipoyl dehydrogenase	13
	TINF05623	Glutamate dehydrogenase	10
	TINF01990	Glutamine synthetase	10
	TINF08380	leucine--tRNA ligase	8
	TINF03369	Glutamate decarboxylase	6
	TINF08349	threonine synthase	6
	TINF01862	Asparagine synthetase	5
	TINF03662	glutamate-5-semialdehyde dehydrogenase	5
	TINF02172	Arg-6 protein	4
	TINF03619	Aconitate hydratase, mitochondrial	3
	TINF06103	Multisynthetase complex auxiliary component p43	3
	TINF03833	Imidazole glycerol phosphate synthase hisHF	3
	TINF03399	Alcohol dehydrogenase, putative	1
	TINF03663	Methionine aminopeptidase 2	1
	TINF00337	Sulfate adenylyltransferase	1

The hub proteins were marked in yellow.

The cell walls of filamentous fungi contain chitin, protein, mannan, and amorphous glucan. Six highly expressed DEPs related to fungal cell walls occurred. Four of these DEPs were involved in the formation of cell wall components and maintaining cell wall integrity. The beta-flanking protein (TINF04729) was a conserved genomic neighbor localized within a recently identified metabolic cell wall gene cluster in genomes of *Aspergillus* spp., which may participate in cell wall biosynthesis (Guerriero et al., 2016). The carbohydrate-binding WSC (TINF05352) was not recognized in substrates but attaches the enzyme to plant and/or fungal cell walls, and may be potentially involved in β-glucan remodeling to maintain cell wall integrity and participate in the stress response (Wawra et al., 2019). The oxidoreductase, 2OG-Fe(II) oxygenase family (putative, TINF05005) may affect the composition of the secondary cell wall (Fang et al., 2012). The sphingolipid long-chain base-responsive protein PIL1 (TINF07009) inhibits protein kinases involved in signaling pathways for cell wall integrity (Delom et al., 2006). The other two DEPs were components of the cell wall and related to different cell wall functions. The chitin-binding, domain 3 (TINF05053) has been predicted to be localized in the cell wall (Zhan and Guo, 2015). Cyanovirin-N (TINF06073) in filamentous ascomycetes is a nonsecretory monodomain protein and a multidomain protein bearing functionally related modules, such as peptidoglycans and chitin-binding domain LysM, in the cell wall (Percudani et al., 2005).

Lipid molecules, such as cholesterol and ergosterol, are found in eukaryotic cell membranes. Eight DEPs were related to membrane and transport. Four of these DEPs were involved in the formation of the plasma membrane. Squalene monooxygenase (TINF07977) is an important enzyme involved in the synthesis of ergosterol, cholesterol, and phytosterols (Zare et al., 2014). The BAR domain protein (TINF01359) is a membrane-shaping protein, which determines organelle biogenesis, membrane trafficking, cell division, and cell migration (Frost et al., 2009). The ankyrin repeats domain-containing protein (TINF00399) is localized on the cytoplasmic membrane during transient expression in onion epidermal cells (Zhang et al., 2010). The oxysterol binding protein (TINF02166) is an important non-vesicular trafficking protein involved in the transportation of lipids in eukaryotic cells, and may also participate in membrane formation (Qiu and Zeng, 2019). Another four DEPs were involved in the formation of membrane organelles, such as vacuoles and vesicles, as well as transportation processes. Carboxypeptidase (TINF00103) has been used as a marker enzyme for investigations on the intracellular transport of vacuolar proteins and vacuolar biogenesis in *S. cerevisiae* (Ohsumi et al., 2001). The hsc70 cochaperone (SGT) (putative, TINF09583) bends membranes based on their ability to oligomerize. This activity promotes endosomal microautophagy and the turnover of specific synaptic proteins (Uytterhoeven et al., 2015). The apolipoprotein/apolipoprotein (TINF08525) is involved in phagocytosis, and possibly pattern recognition (Whitten et al., 2004). The vesicle-fusing ATPase (TINF02607) serves a dual role in vacuolar integrity to regulate vacuole fusion and fission reactions in yeast (Qiu, 2012).

Five DEPs were related to mycelial growth and development. The cipC-like antibiotic response protein (putative, TINF04883) was exclusively found in the hyphal morphotype, which enables invasive growth of *Aspergillus fumigatus* during infection (Bauer et al., 2010). The protein kinase activator (Mob2) (putative, TINF07215) is crucial for normal hyphal development (Gutiérrez-Escribano et al., 2011). Two DEPs targeting the endoplasmic reticulum (ER), the short-chain dehydrogenase/reductase family protein (TINF05550), and signal recognition particle subunit SRP72 (TINF01479) are related to cell death, defense responses, and protein transport (Brown et al., 1994; Zheng et al., 2022). The glia maturation factor beta (TINF07923) is a regulator of the actin cytoskeleton with a unique role in remodeling the actin network architecture, and it has roles in controlling actin filament spatial organization and the dynamics underlying cell motility, endocytosis, and other biological processes (Goode et al., 2018).

### 3.6 Quantitative real-time polymerase chain reaction (PCR) validation of DEP expression

**Figure 8:**
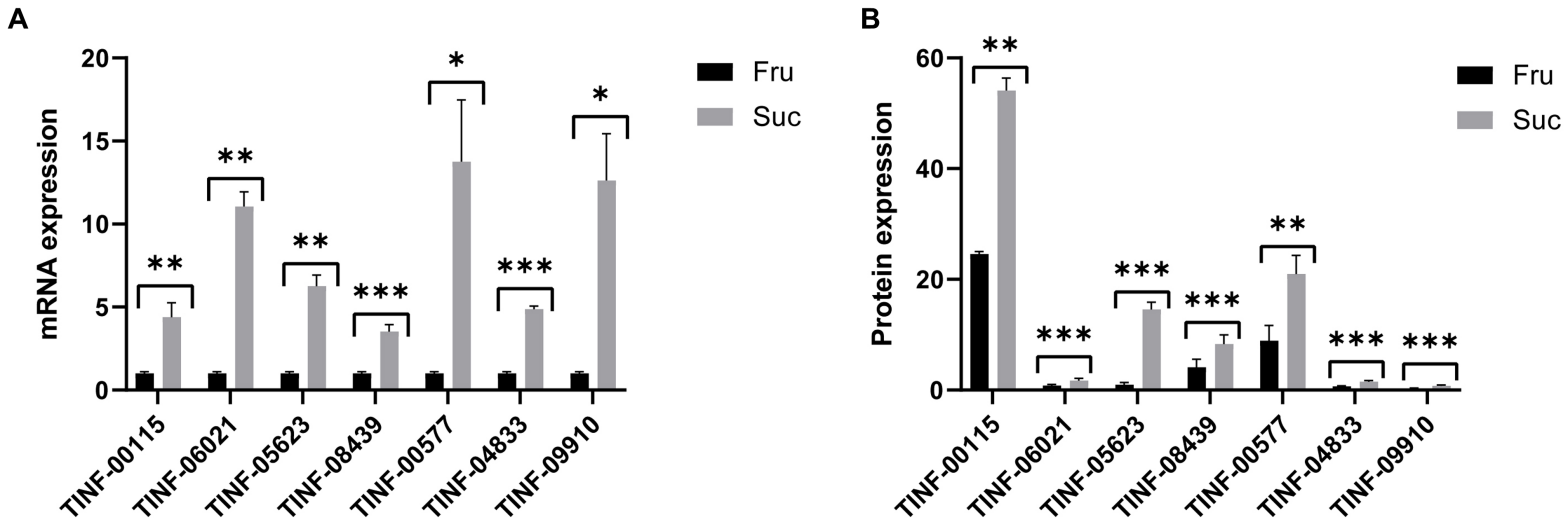
Quantitative real-time PCR validation of the proteomic results. **(A)** The mRNA expression of the seven hub DEPs. **(B)** The protein expression of the seven hub DEPs. ^***^: *p*-value <0.001, ^**^: *p*-value <0.01, ^*^: *p*-value <0.05. The protein expression changes are consistent with those of their mRNAs.

Gene expression is largely consistent with protein expression. We used RT-qPCR to determine the gene expression levels (mRNA content) of seven key DEPs, including the hub DEPs in the pathways of carbohydrate and monosaccharide metabolism, protein-containing complex organization and mRNA binding, amino acid metabolism, (ribose) and phosphorus and purine ribonucleotide metabolism to verify the accuracy of our proteomics results. The primers for RT-qPCR analysis are listed in Supplementary Table S6-1. The results showed that the changes in gene expression (Supplementary Table S6-2) were consistent with changes in protein expression (Supplementary Table S6-3), indicating the reliability of our proteomic results and the accuracy of the DEP screening (Figure 8).

## 4 Conclusion

CsA production and the accumulation of mycelia differed in Fru and Suc media. The difference was most obvious on day 6 of culture when CsA was produced in the Fru medium and mycelium preferentially grew in the Suc medium. The mycelium proteome in these two groups was determined and compared. The highly expressed mycelial proteins cultured in Fru medium were involved in the regulation of CsA production, metabolism, and transportation. These DEPs participated in the processes of small molecules, such as carboxylic acid, oxic acid, and organic acid metabolism; lipid metabolism; catabolic processes of organic substances, particularly organo-nitrogen compounds; CsA transport and exocrine-derived functions of the ER, exosomes, and obsolete cytoplasmic parts; regulating the synthesis of the CsA substrate Bmt; functions of transcriptional regulators. The highly expressed DEPs in the Suc medium were involved in the regulation of mycelial growth. They were mainly involved in the processes of the abandoned REDOX or coenzyme metabolism; small molecules, such as antibiotics, pyridine-containing compounds, amino acids, carbohydrates, biosynthesis or metabolism; response to stress, such as exogenous stimuli/inorganic/chemical stimuli, exogenous catabolism, oxidative stress, and detoxification; genetic information or epigenetic changes, such as changes in nuclear organization and DNA conformation, nucleotide (phosphorylated) metabolism; cell component assembly/organization; cell wall integrity; membrane metabolism and vesicle transport, intramembrane localization, and regulation of filamentous growth.

## Data availability statement

The datasets presented in this study can be found in online repositories. The names of the repository/repositories and accession number(s) can be found in the article/Supplementary material.

## Author contributions

JW: Conceptualization, Data curation, Formal analysis, Investigation, Supervision, Validation, Visualization, Writing – original draft. ML: Conceptualization, Data curation, Formal analysis, Investigation, Supervision, Validation, Visualization, Writing – original draft. CM: Conceptualization, Data curation, Formal analysis, Investigation, Validation, Visualization, Writing – review & editing. SL: Data curation, Formal analysis, Investigation, Validation, Visualization, Writing – review & editing. JZ: Data curation, Formal analysis, Visualization, Writing – review & editing. YF: Conceptualization, Methodology, Writing – review & editing. LG: Conceptualization, Methodology, Writing – review & editing. HY: Conceptualization, Methodology, Writing – review & editing. XY: Conceptualization, Data curation, Formal analysis, Funding acquisition, Investigation, Methodology, Project administration, Resources, Supervision, Validation, Visualization, Writing – original draft, Writing – review & editing.

## Footnotes

^1^ https://cn.string-db.org/cgi/
