# Supplementary material for: Comparative proteomics reveals the mechanism of cyclosporine production and mycelial growth in Tolypocladium inflatum affected by different carbon sources
Source: Front Microbiol. 2023 Dec 8;14:1259101. doi: 10.3389/fmicb.2023.1259101 (PMC10757567; doi:10.3389/fmicb.2023.1259101)
Supplement: Supplementary file 1 [file Data_Sheet_1.zip › Supplementary figures.pdf]

Supplementary figures

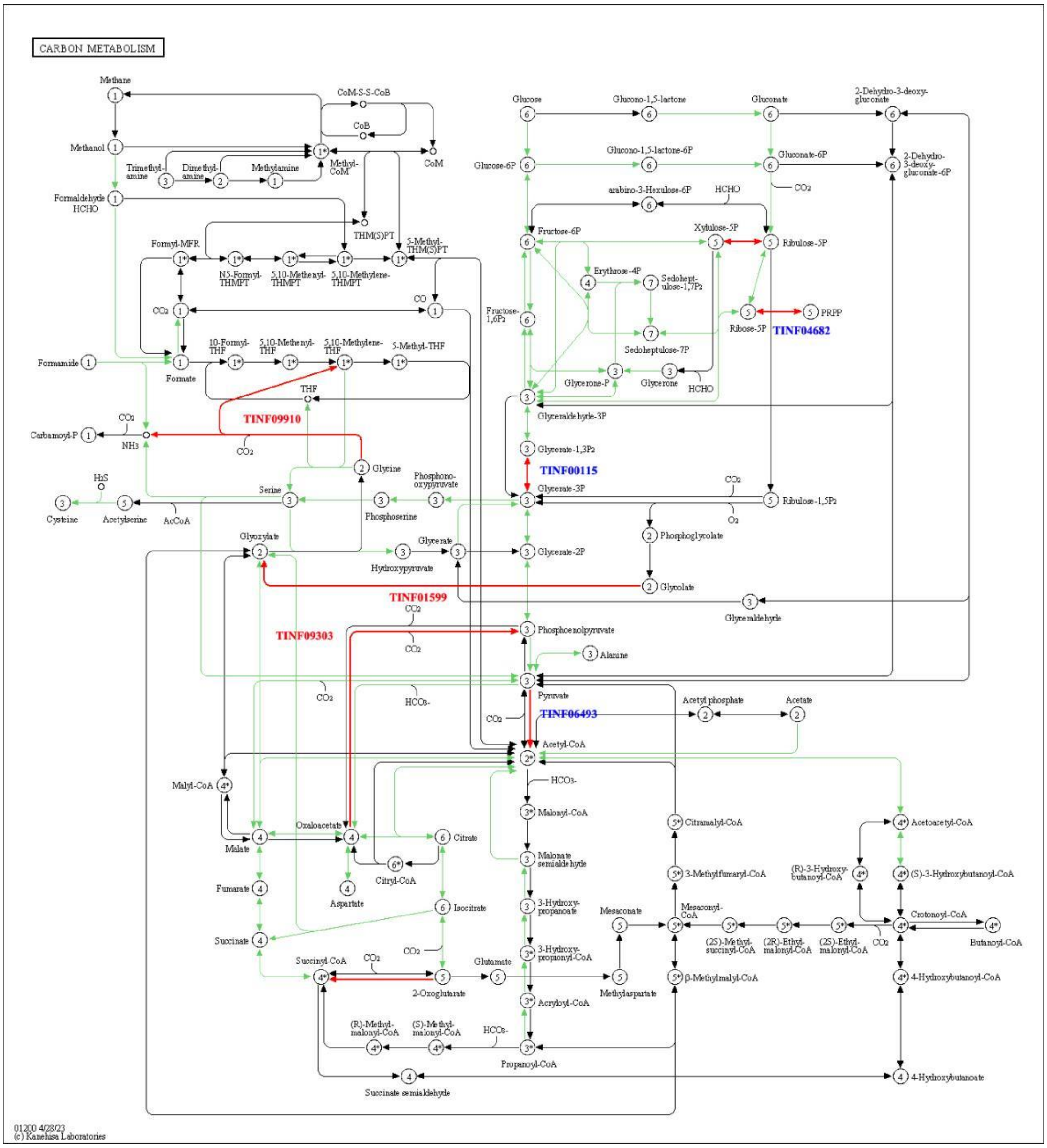

Fig. S1 6 DEPs marked in red and blue were related to carbon metabolism.

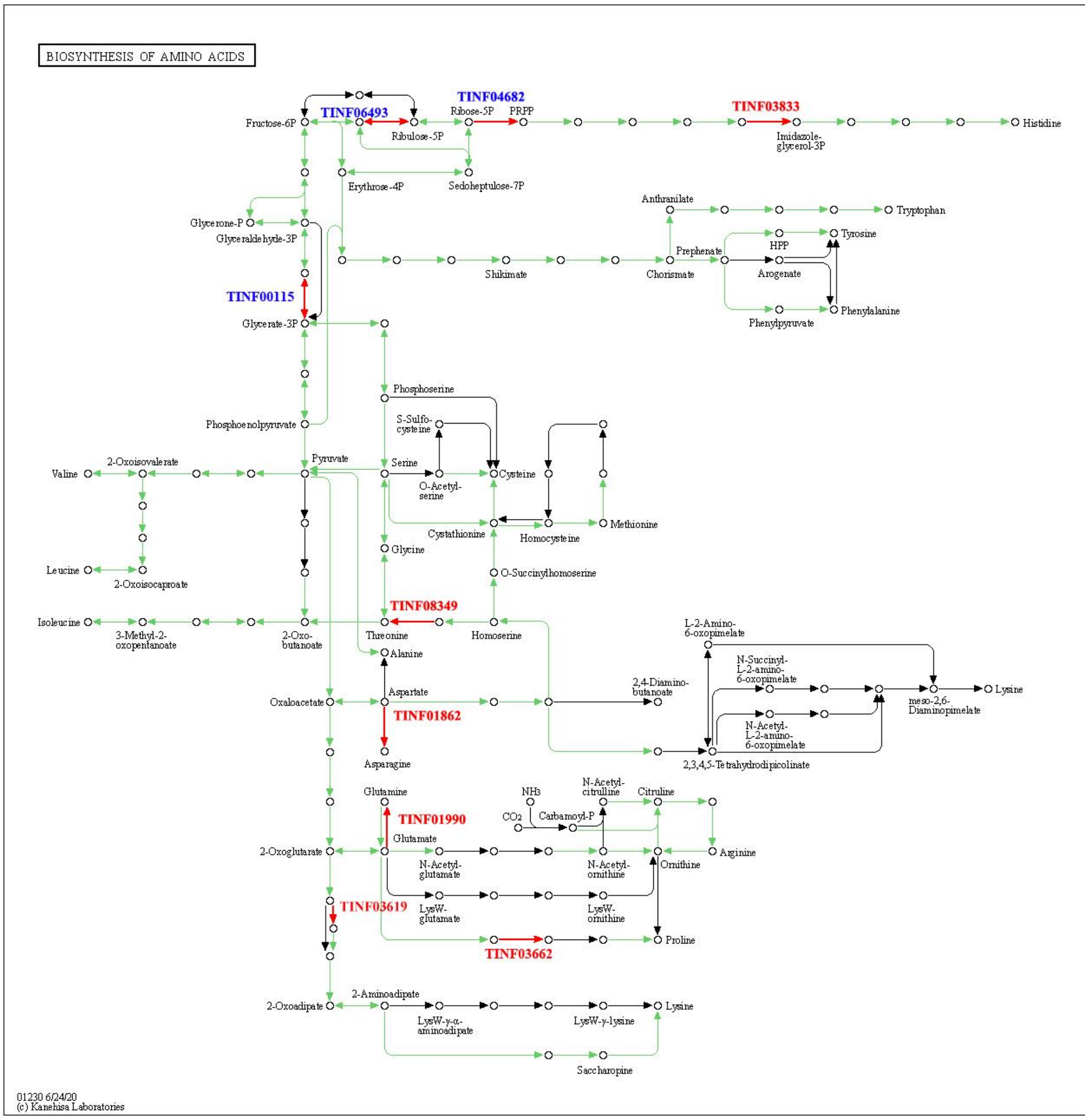

Fig. S2 9 DEPs marked in red and blue related to amino acid biosynthesis. The DEPs both closely related to carbon and amino acid metabolism were blue-colored.

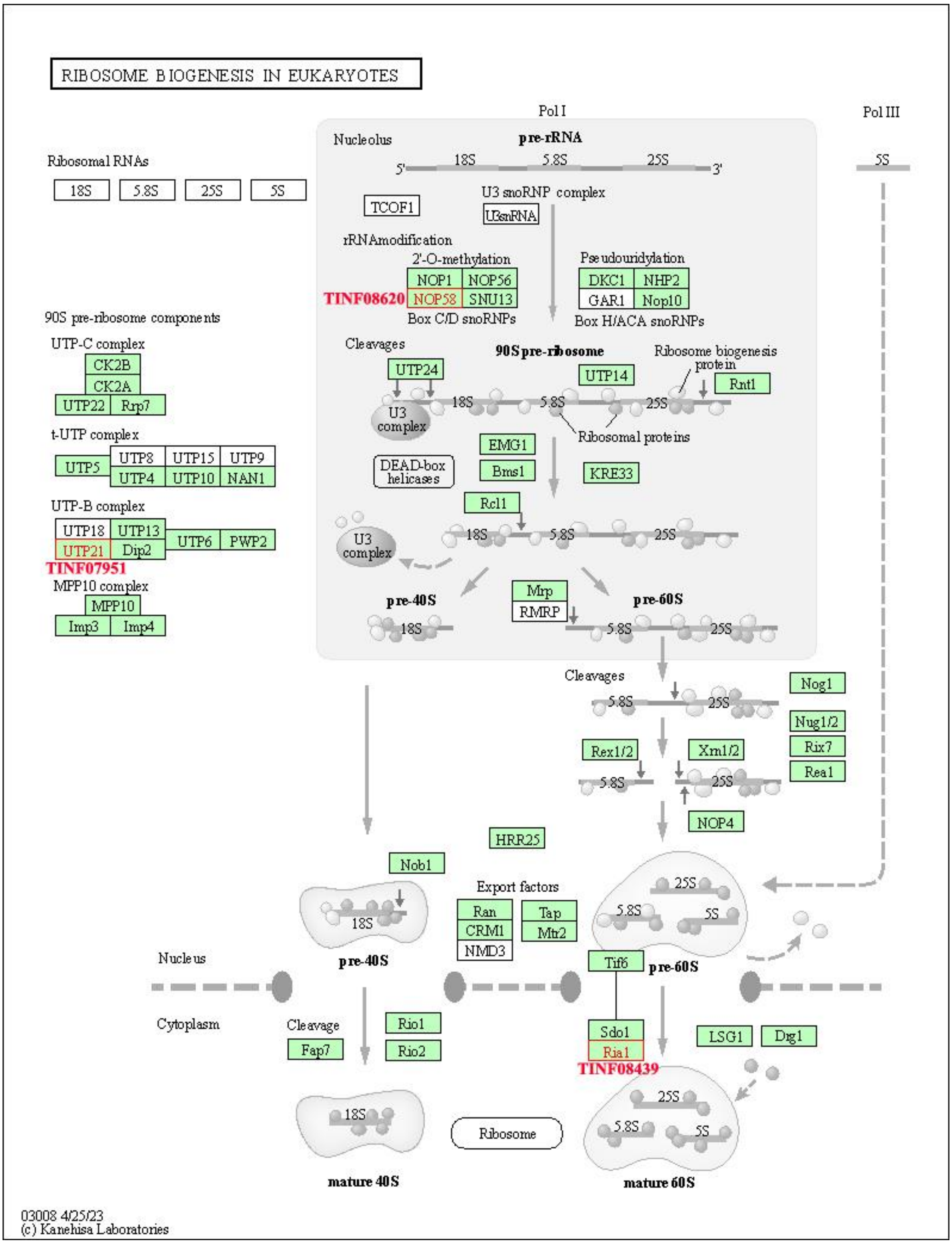

Fig. S3 3 DEPs marked in red related to ribosome biogenesis or function.

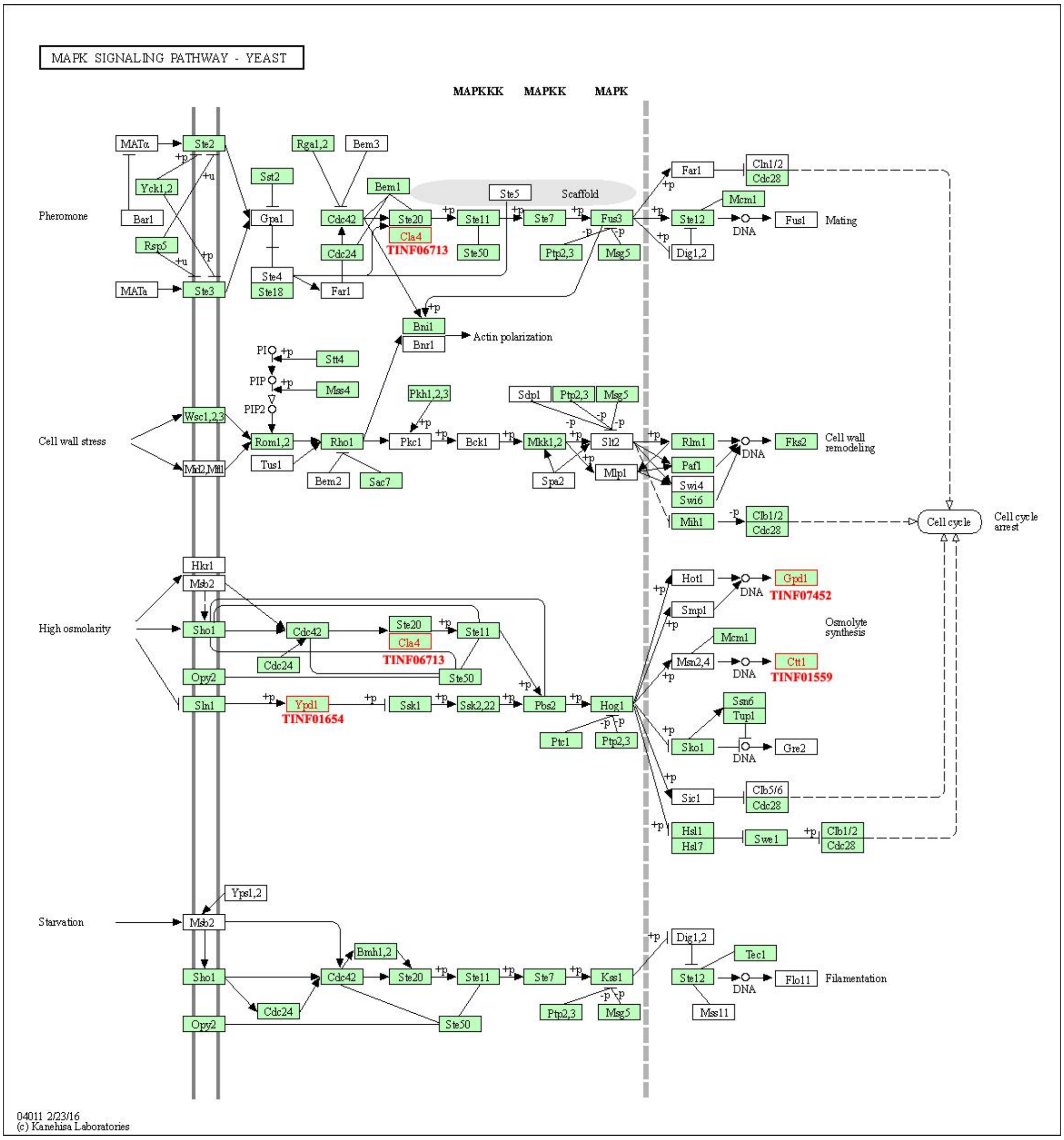

Fig. S4 4 DEPs marked in red related to the MAPK signaling pathway.
